# Brachioradial pruritus secondary to cervical disc protrusion – a case report

**DOI:** 10.1093/jscr/rjac277

**Published:** 2022-08-16

**Authors:** Cezar Octavian Morosanu, Gloria Etim, Andrew Folusho Alalade

**Affiliations:** Department of Neurosurgery, Royal Preston Hospital, Preston, UK; Human Anatomy Resource Centre, Faculty of Health and Life Sciences, University of Liverpool, Liverpool, UK; Department of Neurosurgery, Royal Preston Hospital, Preston, UK; Department of Neurosurgery, Royal Preston Hospital, Preston, UK

## Abstract

Brachioradial pruritus (BRP) is a rare chronic neuropathy of the skin of the arms and forearms that presents with itching, burning or tingling, with no associated dermatological features. Sun exposure and cervical spine pathology have been described as causes for BRP; however, the exact aetiology is often unclear. Herein, we discuss the case of a 63-year-old female patient who presented with BRP with a C5–C6 distribution. Physical examination excluded skin conditions, thus magnetic resonance imaging was done and revealed a C5–C6 disc protrusion. Anterior cervical discectomy and fusion were performed leading to the resolution of symptoms. The case emphasizes the beneficial role of anterior cervical discectomy and fusion as a last resort in patients with refractory pruritus of discogenic cause.

## INTRODUCTION

Brachioradial pruritus (BRP) is a rare form of dysaesthesia that is localized unilaterally or bilaterally to the dorsolateral part of the forearms on the skin overlying the brachioradialis muscle, characterized by persistent chronic itching, tingling, burning and stinging. It can involve the upper arms and shoulders [1] and in some cases can extend to the neck and even lower extremities [2].

BRP was first described in 1968 by Waisman, as a solar pruritus or *pruritus aestivalis*, on the lateral aspect of one or both elbows, corresponding to the insertion of the brachioradialis muscle. The author reported it as a response to prolonged sunlight exposure, with no associated scaling, dermatitis or hyperpigmentation on the skin of the affected area [3].

In 1983, Heyl proposed cervical spine radiculopathy as an aetiology of BRP [4]. Although the initial suggestion of UV light causing BRP was still valid, Heyl emphasized that this was only a contributing factor, and the true cause was in the peripheral nervous system. In the absence of dermatological features, other sources should be investigated, and the treatment must be adapted accordingly. We describe the case of a patient with refractory BRP in which all pharmacological therapies failed and in which surgical decompression was the solution.

## CASE PRESENTATION

A 63-year-old female patient presented to her general practitioner with 5-year history of insidious onset itching in both her forearms, which has been worsening for the past months. She described it as a severe intermittent burning itching sensation, worse on the left side, which lasted for several hours for most days, aggravated by heat and eased by cold, unrelated to any particular physical activity. She was treated with topical steroids, amitriptyline and acupuncture; however, none seem to have made any difference. She was referred to dermatology who felt that the distribution of her pruritus was more consistent with a peripheral nerve pathology rather than a skin issue.

Her medical history included asthma for which she was prescribed salbutamol and beclomethasone and hypothyroidism for which she was taking regular levothyroxine. On previous medical encounters, she was also diagnosed with an intervertebral disc prolapse for which she underwent physiotherapy. She had an adverse reaction to penicillin and trimethoprim, but no specific allergy. She was otherwise well, having a sedentary lifestyle working as a government benefits adviser, did not smoke and had occasional alcohol.

On examination, no significant irritation, erythema, swelling or pigmentation could be identified on the skin of her forearms. Her power was normal in her brachioradialis muscles bilaterally as well as in the other muscle groups. Myotomes, dermatomes and reflexes of the upper and lower limbs were all unremarkable.

**Figure 1:**
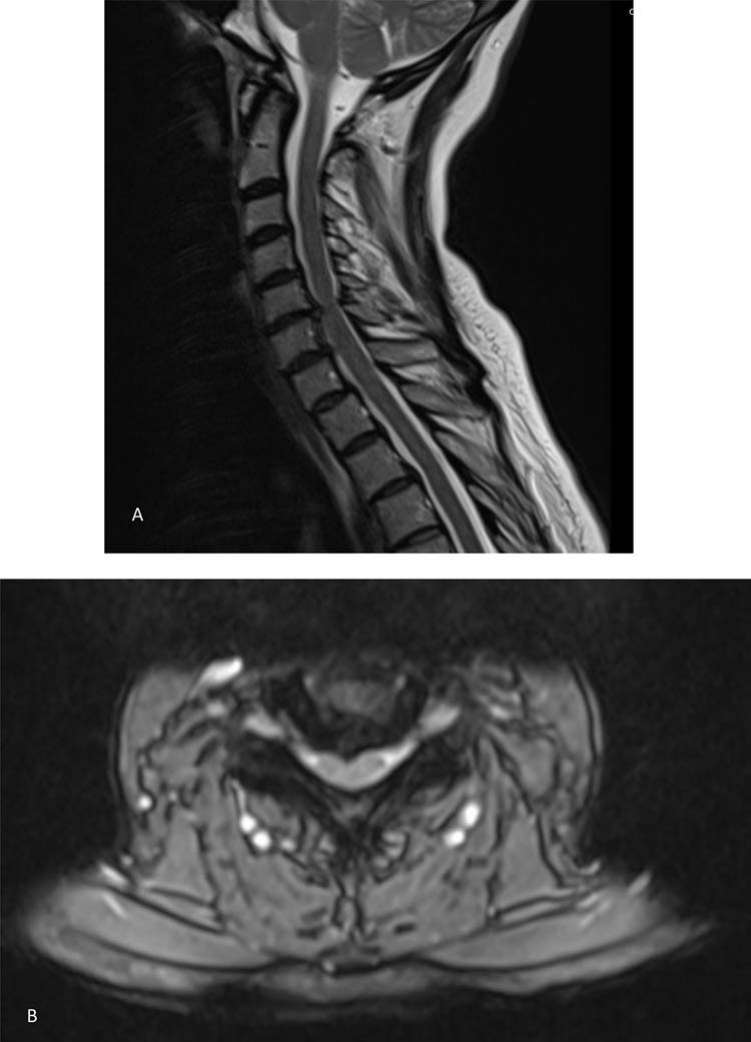
MRI showing bilateral C6 nerve root compression and left C7 nerve compression, C5–C8 disc herniation indenting the ventral aspect of the cord without typical signs of compression myelopathy or significant intramedullary signal abnormality. **A**. Sagittal view. **B**. Axial view.

She was further referred for spinal magnetic resonance imaging (MRI) which revealed a C5–C6 disc compression (Fig. 1). Following the scan, she was further referred to neurosurgery where a discussion was carried out with regards to the risks and benefits of surgery to relieve the pressure on the nerves. Given the thecal compression and the significant pruritus refractory to any therapy, the decision was to proceed with a surgical intervention.

**Figure 2:**
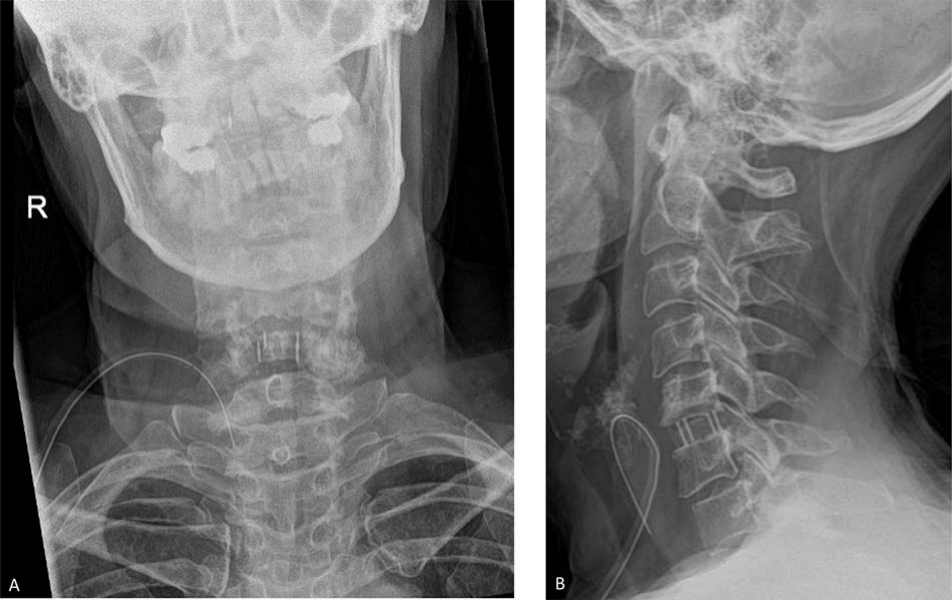
Post-operative radiographs. **A**. Anteroposterior view. **B**. Lateral view. The C5/C6 disc was identified using X-ray guidance and the vertebral bodies were distracted using Caspar pins. The endplates were prepared with a curette and the discectomy was performed. The posterior osteophytes were thinned with a matchstick drill and the posterior longitudinal ligament and posterior osteophytes were removed with Kerrison rongeurs.

The patient underwent a C5/C6 anterior cervical discectomy and fusion (ACDF) (Fig. 2). Following the procedure, she had an uneventful recovery, and she was discharged the following day. At 3-month follow up, her symptoms had resolved entirely, and she resumed her normal work 5 weeks after surgery.

## DISCUSSION

BRP is a condition that has been poorly described in literature. Various causes have been identified to generate this issue; however, there is no consensus as to the best management. As in the case of the T2–T6 interscapular dysaesthesia seen in notalgia paraesthetica [5], BRP is also confined to specific dermatomes, guiding the clinician to investigate the spine as a potential origin of the symptoms. BRP has been reported to occur in cases of cervical degenerative disc pathology [6]; however, the association is variable in literature. Marziniak *et al*. conducted an MRI study on a patient population of 41 individuals with BRP and concluded that 80.5% had intervertebral foramen stenosis and cervical disc nerve compression, leading to a statistically significant correlation with BRP. Conversely, there was no association between BRP and degenerative spinal changes [7]. Goodkin *et al*. found that 50% of their cases with BRP had cervical spinal pathology that was correlated with the dermatomal distribution [8].

Given the variability in aetiology, the treatment is often a matter of debate. Only a small fraction of publications report the results of surgical treatment; however, spinal manipulation is recognized as a solution for this form of refractory BRP. Tait *et al*. describe 10 out of 14 patients with BRP who had a resolution of symptoms after surgery [9]. Binder *et al*. described a 64-year-old case who underwent a ventral spinal fusion with a cage implantation for refractory BRP secondary to C6 nerve compression [10]. In addition, Salzmann *et al*. describe the case of 56-year-old who had a disabling pruritus in both arms secondary to multilevel cervical spondylosis and foraminal stenosis for which ACDF was the only solution [11].

The pathophysiology behind BRP is unclear. A distinction can potentially be made between discogenic BRP and photogenic BRP. Although there is evidence to suggest spinal disease can be incriminated for this dermatomal manifestation, some authors like Orton *et al*. advocate for ultraviolet radiation from sun exposure as the culprit [12]. A reduction in cutaneous innervation has also been identified on histology in some patients [13] as well as a non-specific myelin sheath splitting in some studies [14]. Nerve damage due to sun exposure and disc compression/ degeneration are possibly complementary and the combination of the two can lead to the clinical picture seen in some patients.

## CONCLUSION

BRP is an uncommon pathology, and its best management remains a matter of controversy. Physicians should be aware of the potential discogenic nature of this condition to investigate the patient for cervical spinal compression with adequate imaging from an early stage. Our case highlights the impact BRP can have on patients and the need to consider surgery (in the form of decompression) in cases in which symptomatic relief is not achieved with conventional measures.
